# GPR44-Dependent Regulation of the Selenoproteome by eIF4a3 in Murine Acute Myeloid Leukemia-Initiating Stem Cells

**DOI:** 10.1007/s12011-025-04724-9

**Published:** 2025-06-27

**Authors:** Deborpita Sarkar, Fenghua Qian, Robert F. Paulson, K. Sandeep Prabhu

**Affiliations:** Department of Veterinary and Biomedical Sciences, Center for Molecular Immunology and Infectious Disease and Center for Molecular Toxicology and Carcinogenesis, The Pennsylvania State University, University Park, PA 16802 USA

**Keywords:** Cyclopentenone prostaglandins, Selenium, Selenoprotein hierarchy, PTGDR2, GPCR, MRNA stability

## Abstract

Acute myeloid leukemia (AML) remains an aggressive hematologic malignancy, with leukemia-initiating stem cells (LICs) playing a critical role in disease progression and therapeutic resistance. In this study, we investigated the role of GPR44, a G-protein coupled receptor of arachidonic acid-derived prostaglandin D_2_ (PGD_2_) and its cyclopentenone prostaglandins (CyPGs) metabolites, Δ^12^-PGJ_2_ and 15d-PGJ_2_, in regulating selenium metabolism and selenoprotein expression in AML LICs. Transplantation of *Gpr44*^*−/−*^ LICs into donor mice led to aggressive leukemogenesis. Transcriptomic and proteomic analyses revealed that GPR44 deletion significantly altered the selenoproteome, with downregulation of Txnrd1, Txnrd3, Selenop, and Gpx2, while upregulating Gpx3, Gpx4, Selenoo, and Msrb1. These findings suggest that GPR44 influences redox homeostasis and leukemic cell survival by modulating selenium utilization. Notably, increased expression of eIF4a3 in *Gpr44*^*−/−*^ LICs suggested a potential mechanism for selective selenoprotein repression through SECIS-binding protein 2 (SBP2) inhibition. Additionally, upregulation of SBP2 and selenophosphate synthetase 2 (SPS2) indicated an adaptive response to maintain selenium incorporation. Given the role of selenium in redox balance, metabolism, and immune function, targeting selenium metabolism in GPR44-expressing AML may offer a novel therapeutic approach. Our findings reveal a previously unrecognized link between GPR44 signaling, selenium metabolism, and leukemia progression, warranting further studies to explore selenoprotein-targeting strategies for AML treatment.

## Introduction

Acute myeloid leukemia (AML) is a hematologic malignancy marked by the rapid proliferation of undifferentiated myeloid cells, leading to impaired hematopoiesis, cytopenia, and severe complications [1]. AML is genetically and phenotypically heterogeneous, arising from genetic and epigenetic alterations that block normal differentiation, causing leukemic blast accumulation [2]. Despite advancements in chemotherapy, targeted therapies, and bone marrow transplantation, relapse remains a major challenge, necessitating novel treatment. A key factor in AML persistence is leukemia-initiating stem cells (LICs), a rare subset of self-renewing leukemic stem cells (LSCs) driving leukemogenesis, chemoresistance, and disease relapse [3]. LICs can self-renew and generate heterogeneous leukemic populations sustaining the disease [4]. In experimental AML models with MLL-AF9 translocation (t9:11), LICs display dysregulated transcriptional and metabolic programs enhancing survival and proliferation [5–7]. Targeting LICs is crucial for disease remission.

The human and mouse selenoproteome consists of 25 and 24 selenoproteins, respectively, many acting as antioxidant enzymes, including glutathione peroxidases (GPX1-4), thioredoxin reductases (Txnrd1-3), selenoprotein P (Selenop), and selenoprotein W (Selenow) [8, 9]. These proteins reduce reactive oxygen species (ROS), regulate signaling pathways, and protect cellular components from oxidative damage. Selenoprotein synthesis involves a unique translational mechanism requiring the incorporation of selenocysteine (Sec), the 21^st^ amino acid, through the Sec insertion sequence (SECIS) in the 3′ UTR of mRNAs [10]. SECIS-binding protein 2 (SBP2) recognizes this secondary structure element to decode UGA codon for Sec incorporation [11]. Selenophosphate synthetase 2 (SPS2) is essential for selenophosphate biosynthesis [12]. eIF4a3, a DEAD-box RNA helicase, functions in mRNA surveillance, nonsense-mediated decay (NMD), and translational regulation [13]. It contributes to cancer progression by modulating mRNA stability and translation [13]. eIF4a3 has been shown to suppress SELENOF translation by interfering with its SECIS element, thereby limiting its protein expression. This regulatory mechanism may play a role in prostate cancer progression, as decreased SELENOF levels have been linked to tumor development [14]. eIF4a3 interacts with SECIS elements, potentially inhibiting SBP2 binding and selectively repressing selenoprotein translation [14], a key mechanism thought to underlie hierarchical selenoproteome expression.

Altered selenium metabolism has been reported in AML, with deficiency linked to increased cancer risk and supplementation showing chemopreventive effects [15]. Selenium levels correlate with leukemia severity, and selenium-based compounds induce apoptosis in leukemic cells [15]. However, the precise role of selenium in AML, particularly in LIC function, remains unclear. Using a murine AML model driven by MLL-AF9, we reported that dietary selenium supplementation with 0.4 ppm sodium selenite led to significant disease remission and survival via prostaglandin D_2_ (PGD_2_)-derived cyclopentenone prostaglandins (CyPGs), Δ^12^-PGJ_2_ and 15d-PGJ_2_ [7]. These endogenous lipid mediators activated GPR44, a high-affinity G-protein coupled receptor (GPCR) for PGD_2_ and its CyPG metabolites [16], in LICs, negatively regulating leukemia progression by inducing LIC apoptosis [7]. To investigate the underlying mechanisms, based on the previously published RNAseq dataset [7], eIF4a3 expression in *Gpr44*^−/−^ AML LICs was significantly upregulated compared to their WT counterparts. However, despite the upregulation in SBP2 and SPS2, only a subset of selenoproteins was affected in the *Gpr44*^−/−^ AML LICs. AML progression is driven by LICs with dysregulated metabolic and transcriptional programs. Our studies suggest that selenium metabolism and hierarchical selenoproteome expression probably influence leukemia biology, highlighting vulnerabilities in antioxidant defense and homeostasis. The role of GPR44 in modulating selenoprotein synthesis in AML LICs could assist in uncovering new regulatory networks in selenoprotein expression for potential therapies.

## Results

### Differentially Regulated Selenoprotein Genes in Gpr44^−/−^ AML LICs

**Fig. 1:**
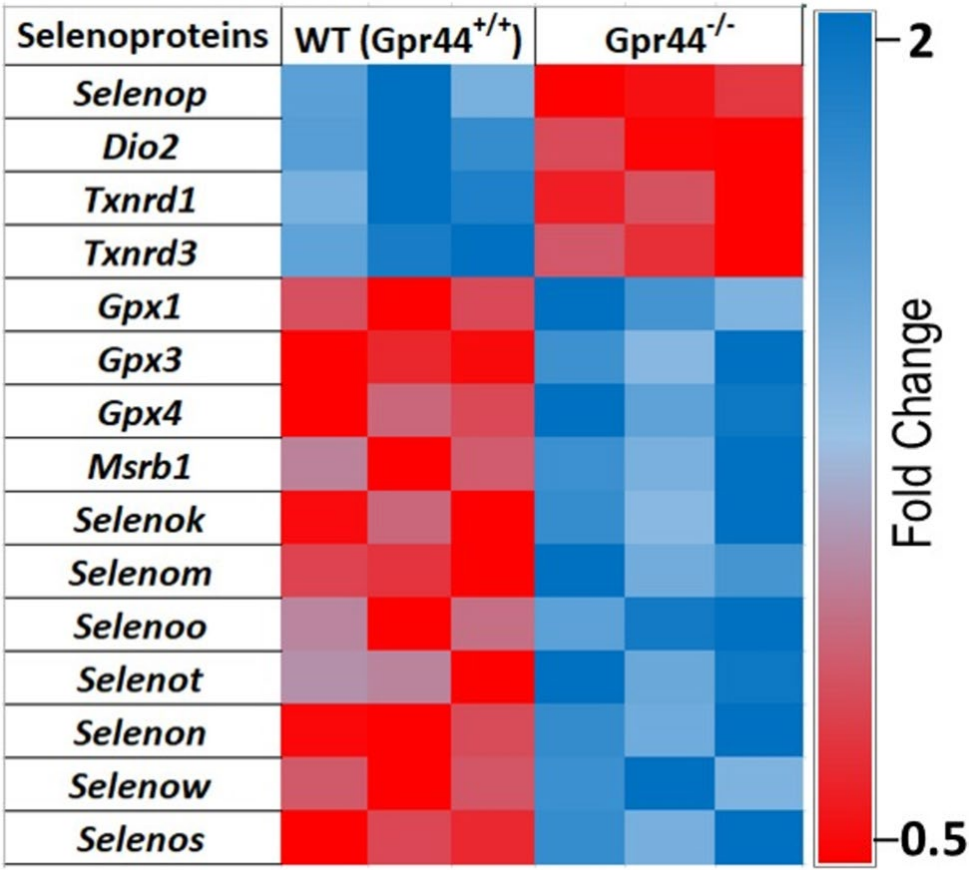
Heatmap of selenoprotein genes in WT (*Gpr44*^+*/*+^) and *Gpr44*^*−/−*^ LICs: this heatmap displays the relative expression levels of various selenoprotein genes showing significant differential expression between wild-type (*Gpr44*^+*/*+^) and GPR44 knockout (*Gpr44*^*−/−*^) primary AML LICs from RNA Seq data. Purified LICs were used for RNAseq experiments as described 7. Plots were generated from normalized read counts. The mean of normalized read counts of *Gpr44*^+*/*+^ was divided by the mean normalized read count of the *Gpr44*^*−/−*^ to derive the fold change. The color scheme denoting fold changes is provided. A multiple *t*-test was applied

In the murine model of AML where LICs expressing MLL-AF9 were transplanted into recipient mice, we observed that deletion of *Gpr44* in LICs led to aggressive AML, supporting the notion that GPR44 has a tumor suppressor role [7]. To investigate the impact of GPR44 deletion on the selenoproteome, RNA sequencing was performed on WT (*Gpr44*^+/+^) and *Gpr44*^−/−^ LICs isolated from mice as described [7]. Figure 1 shows the heatmap illustrating the differential expression of selenoproteins in WT and *Gpr44*^−/−^ LICs. Notably, *Dio2*, *Selenop*, *Txnrd1*, and *Txnrd3* were downregulated in *Gpr44*^−/−^ LICs. On the other hand, *Gpx1*, *Gpx3*, *Gpx4*, *Msrb1*, *Selenok*, *Selenom*, *Selenon*, *Selenot*, *Selenon*, *Selenow*, and *Selenos* were all upregulated in the *Gpr44*^−/−^ LICs. These findings indicate that deletion of GPR44 significantly alters selenoprotein expression profiles in AML LICs.

### Analysis of Selenoprotein Expression and Selenium Incorporation Machinery in Gpr44^−/−^ LICs

**Fig. 2:**
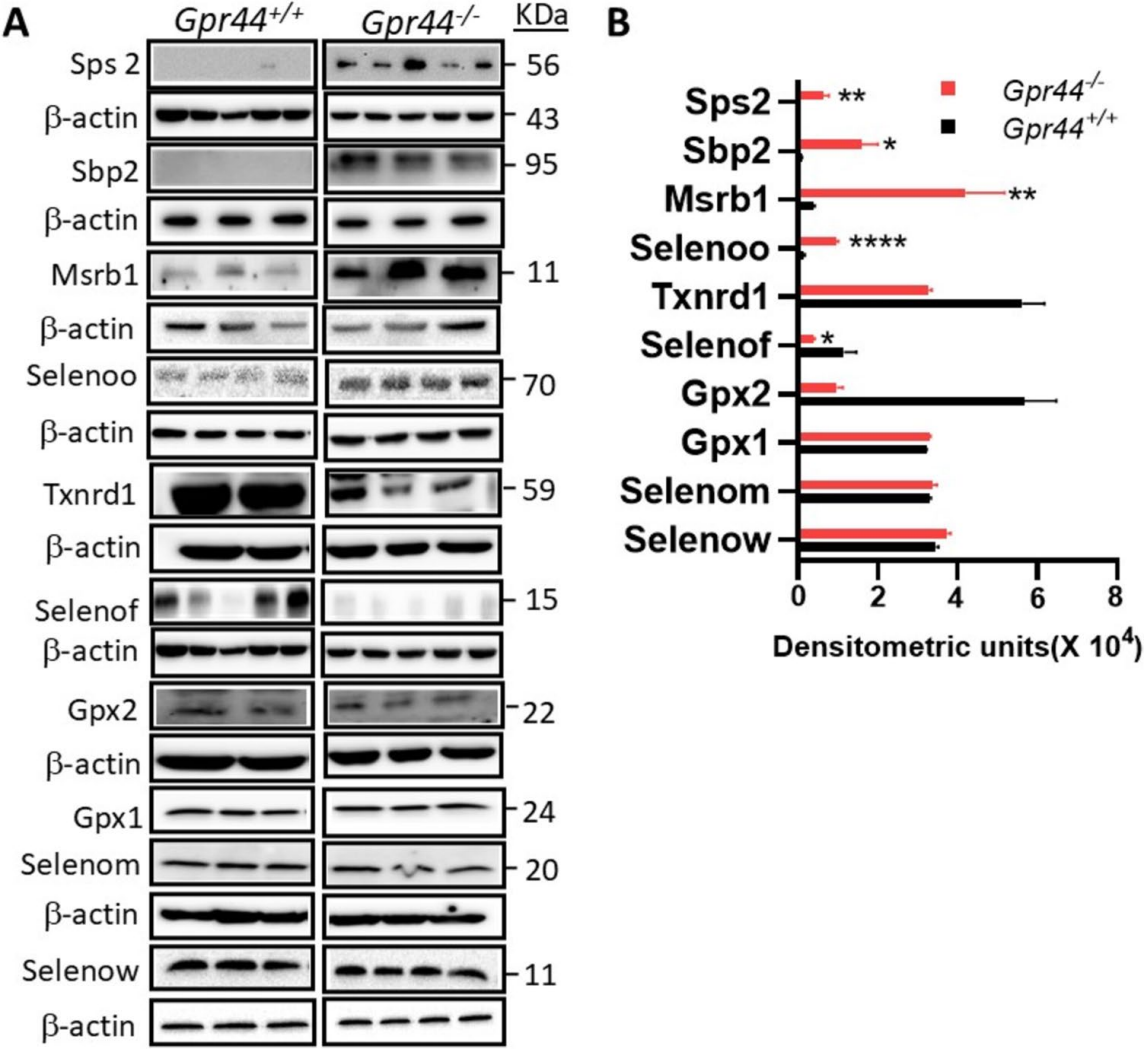
Western blot analysis of selenoprotein expression in WT (*Gpr44*^+*/*+^) and *Gpr44*^*−/−*^ LICs: **A** representative Western blot images show protein expression levels of various selenoproteins in WT (*Gpr44*^+*/*+^) and *Gpr44*^*−/−*^ AML LICs (*n* = 2–5 per genotype). Protein bands corresponding to each target protein are shown. **B** Densitometric quantification of Western blot bands was performed using ImageJ, followed by statistical analysis using multiple *t*-tests. Red and black bars indicate *Gpr44*^*−/−*^ and *Gpr44*^+/+^ LICs, respectively. The data are presented as mean (of *n* =  ≥ 3) ± SEM, with significant differences indicated (*p < 0.05, **p < 0.01, ****p < 0.0001)

To further examine if the impact of deletion of GPR44 on the transcriptional regulation of the selenoprotein genes was sustained even at the protein level, we analyzed their expression in WT and *Gpr44*^−/−^ LICs using Western immunoblots. Western immunoblots revealed differential expression patterns among various selenoproteins in the *Gpr44*^−/−^ LICs when compared to WT LICs. Specifically, Gpx2, TR1, and Selenof were downregulated in *Gpr44*^−/−^ LICs relative to WT counterparts. On the other hand, Selenoo and Msrb1 were upregulated in the *Gpr44*^−/−^ LICs. Interestingly, select few selenoproteins, including Gpx1, Selenom, and Selenow, showed no significant change in expression as a function in *Gpr44*^−/−^ LICs. In addition to these alterations in selenoprotein expression, we also observed the upregulation of key components involved in translational incorporation of Sec into proteins. Both SBP2 and SPS2 were significantly upregulated in *Gpr44*.^−/−^ LICs (Fig. 2A & B). These findings suggest that deletion of GPR44 not only modulates the expression of specific selenoproteins but also enhances the machinery responsible for the incorporation of Sec into these select few proteins in the LICs.

### Upregulation of eIF4a3 and Its Potential Role in Modulating Selenoprotein Synthesis in Gpr44^−/−^ LICs

**Fig. 3:**
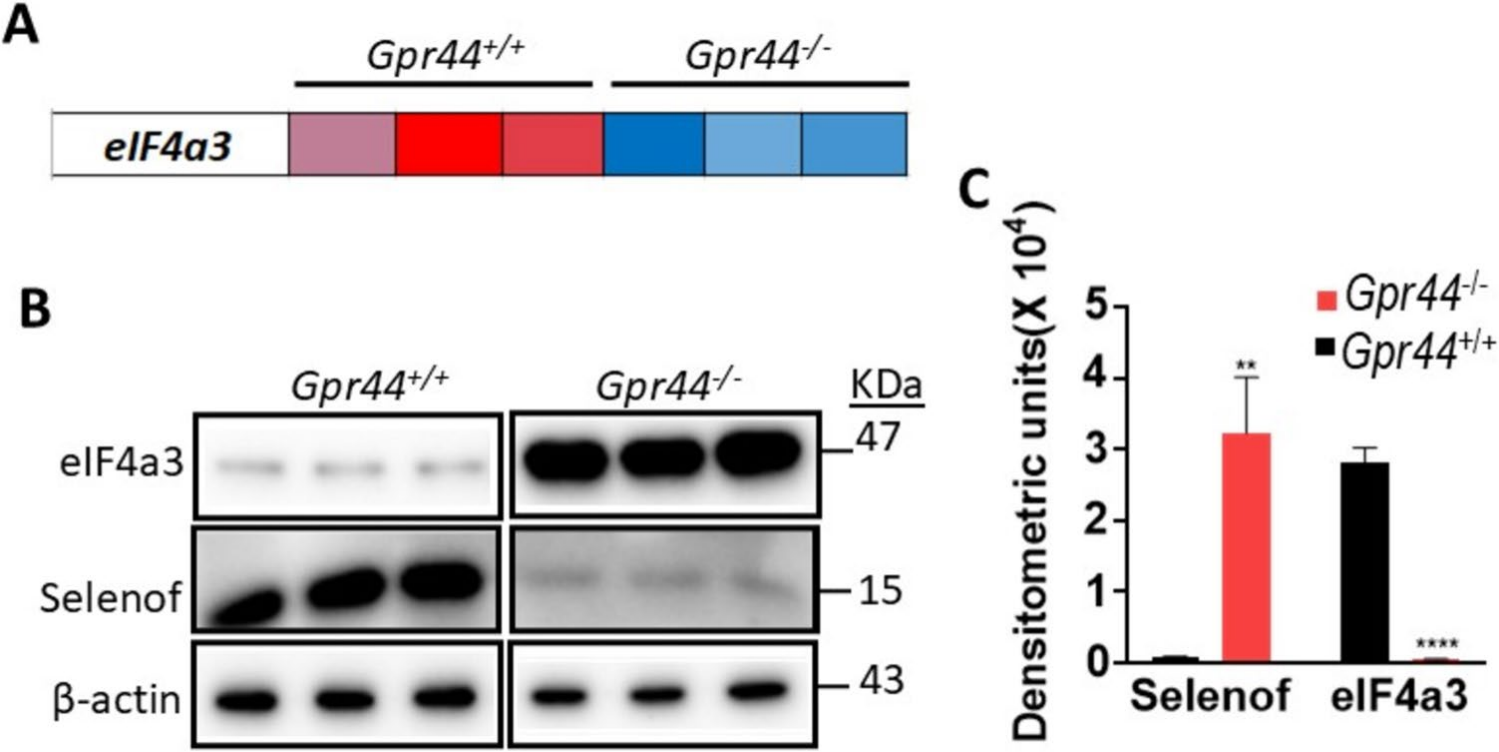
eIF4a3 expression analysis in *Gpr44*^+*/*+^ and *Gpr44*^*−/−*^ LICs: **A** RNAseq transcript values for *eIF4a3* in *Gpr44*^+*/*+^ and *Gpr44*^*−/−*^ LICs. The heatmap represents relative expression levels of *eIF4a3* showing significant differential expression between wild-type (*Gpr44*^+*/*+^) and *Gpr44*^*−/−*^ AML LICs from RNAseq dataset with red and blue indicating low to high expression in the respective genotypes as in Fig. 1. **B** Western blot analysis of eIF4a3 protein levels in purified *Gpr44*^+*/*+^ and *Gpr44*^*−/−*^ AML LICs. Protein bands corresponding to eIF4a3 expression are shown. **C** Densitometric quantification of Western blot bands was performed using ImageJ, followed by statistical analysis using multiple *t*-tests. Red and black bars indicate *Gpr44*^*−/−*^ and *Gpr44*.^+/+^ LICs, respectively. Data are represented as mean ± SEM, with significant differences indicated (**p < 0.01, ****p < 0.0001)

To further elucidate the regulatory mechanisms underlying such a hierarchical regulation of selenoprotein synthesis in *Gpr44*^−/−^ LICs, we reexamined the RNA sequencing dataset [7]. Intriguingly, we uncovered a significant upregulation in eukaryotic initiation factor 4a3 (*eIF4a3*) in *Gpr44*^−/−^ LICs compared to WT LICs (Fig. 3A). Notably, this increase in *eIF4a3* expression was also confirmed at the protein level using Western immunoblot (Fig. 3B). A threefold increase in eIF4a3 was seen in the *Gpr44*^−/−^ LICs along with a 2.8-fold decrease in Selenof in the *Gpr44*^−/−^ LICs when compared to WT LICs (Fig. 3C). Based on the literature, the observed upregulation of eIF4a3 in *Gpr44*^−/−^ LICs suggests uncharted mechanisms in the regulation of selenoprotein expression in these cells. Our findings suggest that the differential expression pattern of selenoproteins observed in *Gpr44*^−/−^ LICs (as in Fig. 2) could highlight the hierarchical regulation of selenoprotein synthesis by GPR44 as well as underscore the role of GPR44 in the complex interplay between eIF4a3 and SBP2.

## Discussion

AML is an aggressive hematologic malignancy, where LICs play a critical role in disease progression and therapeutic resistance [2]. In our study, we investigated the role of GPR44, a high-affinity GPCR for PGD_2_ and CyPGs, in the regulation of selenium and/or selenoprotein expression in AML LICs. Our findings suggest that increased leukemia aggressiveness, accompanied by significant alterations in the selenoproteome at both the transcriptomic and proteomic levels, is regulated by downstream mechanisms driven by GPR44. These alterations highlight a novel regulatory axis involving selenium and PGD_2_ metabolism via the regulation of selenoprotein expression that could have potential implications in AML progression and treatment.

Transcriptomic analysis of AML LICs revealed that several selenoproteins were differentially expressed in *Gpr44*^−/−^ LICs. Specifically, *Dio2*, *Selenop*,* Txnrd1*, and* Txnrd3* were significantly downregulated, while *Gpx1*,* Gpx3*,* Gpx4*,* Msrb1*,* Selenok*,* Selenom*,* Selenoo*,* Selenot*,* Selenon*,* Selenow*, and* Selenos* were upregulated. These findings suggest that GPR44 influences selenium metabolism and incorporation, potentially affecting redox homeostasis, cell signaling, and leukemic cell survival. TXNRD3 plays a crucial role in maintaining cellular redox balance by regulating thioredoxin-dependent antioxidant pathways [17]. The observed downregulation of *Txnrd1* and *Txnrd3* in *Gpr44*^−/−^ LICs suggests an impaired redox defense system, which may contribute to increased oxidative stress and leukemia progression. Conversely, the upregulation of *Gpx1*, *Gpx3*, and *Gpx4* in *Gpr44*^−/−^ LICs suggests a compensatory mechanism aimed at counteracting oxidative stress given that Gpxs are critical for detoxifying reactive oxygen species (ROS) and maintaining intracellular redox balance [18]. The increased expression of these enzymes may enable *Gpr44*^−/−^ LICs to adapt to oxidative stress and sustain their proliferative and self-renewal capacities.

To confirm if the hierarchical expression of selenoproteins at the transcriptomic level was consistent through the subsequent translational steps, we analyzed their steady-state expression at the protein level in *Gpr44*^−/−^ LICs and WT LICs. Notably, even though RNAseq experiments did not detect any appreciable levels of *Gpx2* and *Selenof* mRNA, Gpx2, Txnrd1, and Selenof were detected at the protein level but were downregulated, while Selenoo and Msrb1 were upregulated in *Gpr44*^−/−^ LICs. Interestingly, Gpx1, Selenom, and Selenow exhibited no significant changes in expression, indicating that the deletion of GPR44 selectively impacts selenoproteins. The downregulation of Txnrd1 and Gpx2 leads us to hypothesize that GPR44 could be an important player in redox homeostasis that needs to be experimentally evaluated. TXNRD1 is a key component of the thioredoxin system, which plays an essential role in DNA synthesis, repair, and cell survival [19]. Its reduced expression may impair leukemic cell viability and increase susceptibility to oxidative damage, which was reported to be the case in the LICs from an analogous model of murine CML that we previously reported [20]. Additionally, the downregulation of Selenof, which is involved in protein folding and ER stress response, may contribute to altered protein homeostasis in WT (*Gpr*^+/+^) AML LICs [8]. On the other hand, upregulation in Selenoo and Msrb1 suggests that these selenoproteins may play a protective role in *Gpr44*^−/−^ LICs. Selenoo has been implicated in mitochondrial function and metabolic regulation, which are critical for cancer cell survival [21]. Msrb1, a methionine sulfoxide reductase, helps repair oxidized proteins to maintain optimal function under conditions of oxidative stress [22]. These findings indicate that LICs may adapt to GPR44 deletion by regulating specific selenoproteins that enhance their survival and metabolic plasticity. Regardless, further studies are essential to confirm their biological significance to understand their implications on leukemogenesis.

To further explain the underlying molecular mechanisms in hierarchical selenoprotein expression, we analyzed the regulatory pathways involved in selenoprotein synthesis. Our transcriptomics dataset identified a significant upregulation of eIF4a3 in *Gpr44*^−/−^ LICs that was also confirmed at the protein level, suggesting its role in modulating selenoprotein synthesis. eIF4a3 binds to SECIS elements, thereby repressing SBP2-dependent selenoprotein translation [23]. Increased levels of eIF4a3 in *Gpr44*^−/−^ LICs suggest that eIF4a3 may enhance its interaction with SECIS elements, thereby inhibiting SBP2 binding and selectively repressing some the synthesis of certain selenoproteins, such as Selenof (Fig. 3B). Such a selectivity regulated by GPR44 is novel, which could explain the differential expression patterns and hierarchy observed in our study, clearly highlighting the indispensable nature of some selenoproteins. Interestingly, we also observed an increased expression of key Sec incorporation machinery components, including SBP2 and SPS2. SBP2 is a critical factor for the translation of selenoproteins, while SPS2 is involved in selenophosphate synthesis [11]. The upregulation of these proteins suggests that *Gpr44*^−/−^ LICs may enhance their capacity for selenium utilization, potentially to compensate for the selective repression imposed by increased eIF4a3.

**Fig. 4:**
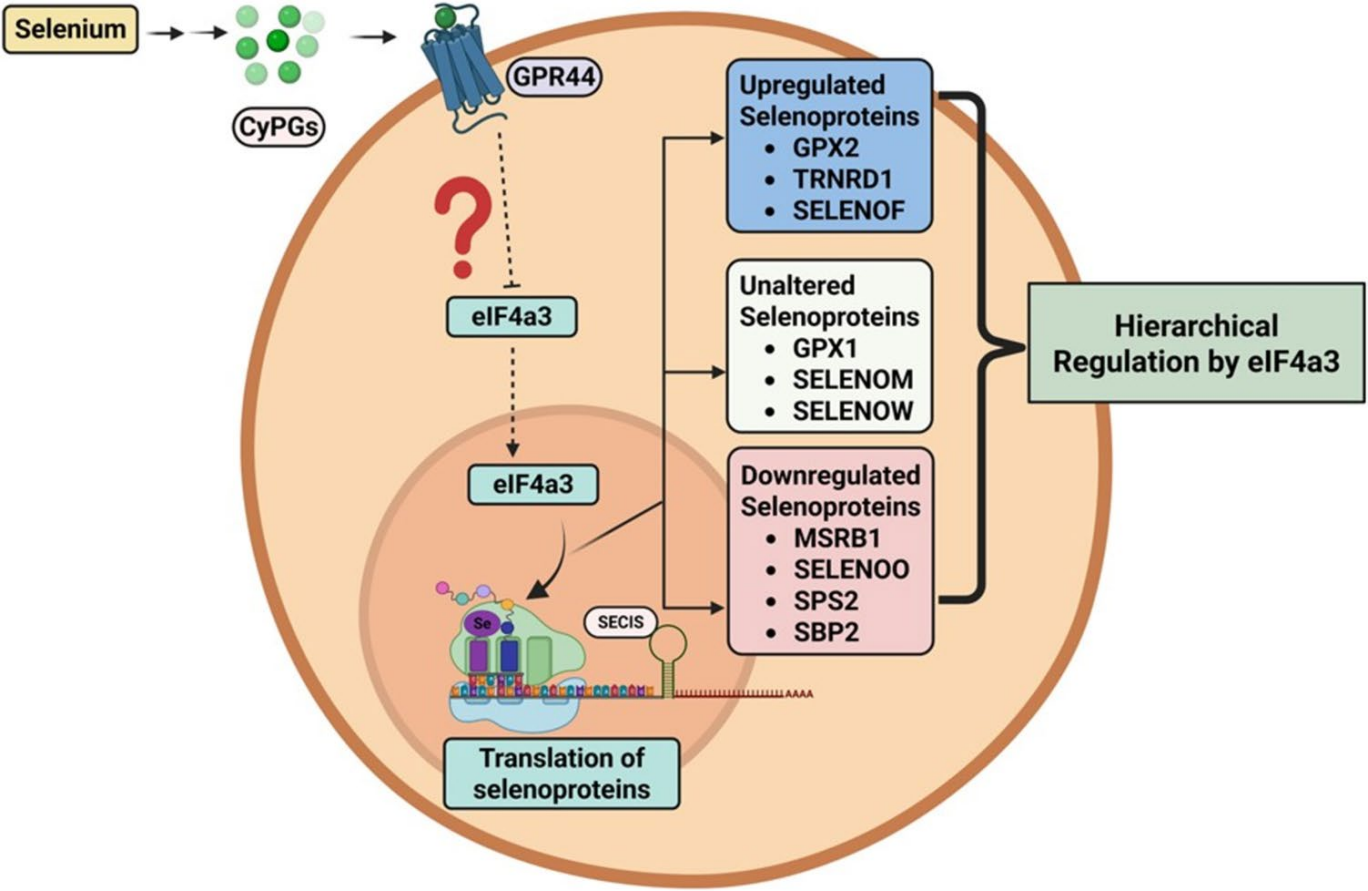
Model of GPR44-mediated regulation of selenoprotein translation via eIF4a3 in AML LICs: Schematic illustration of the potential regulatory pathway linking selenium metabolism, GPR44 signaling, and hierarchical selenoprotein translation through eIF4a3. Selenium availability leads to the production of endogenous CyPGs, which activate GPR44. GPR44 activation influences the expression and activity of eIF4a3, a known regulator of mRNA stability and translation. eIF4a3 modulates the translation of selenoproteins through its interaction with the SECIS element and other proteins of the selenoprotein synthesis machinery. The hierarchical regulation of selenoproteins by eIF4a3 results in distinct expression patterns: upregulation of Gpx2, Txnrd1, and Selenof; unaltered expression of Gpx1, Selenom, and Selenow; and downregulation of Msrb1, Selenoo, SPS2, and SBP2. Notably, SPS2 and SBP2 play critical roles in selenoprotein biosynthesis, suggesting that their downregulation may contribute to selective alterations in the selenoproteome. These findings indicate a complex and uncharted regulatory mechanism connecting GPR44 signaling to eIF4a3 dependent selenoprotein regulation, potentially impacting leukemic cell survival and function

Our findings highlight a novel interplay between GPR44 signaling, selenium metabolism, and selenoprotein synthesis in AML LICs. The observed alterations in selenoprotein expression and selenium incorporation suggest that GPR44 deletion in LICs rewires cellular redox and metabolic networks, contributing to aggressive disease. GPR44 expression is modulated in AML patients, as shown by TCGA data [24], where decreased expression shows lesser survival, suggesting its role in AML pathophysiology. While *GPR44* expression varies across cancers (data not shown), GPR44 could have broader clinical significance via influencing selenoprotein expression, as in AML LICs. Targeting the selenium pathway in GPR44-expressing cancers could offer a novel therapeutic strategy. Several studies have demonstrated the potential of selenium-based compounds in cancer therapy, particularly in modulating oxidative stress and apoptosis [25–27]. Our data suggest that manipulating selenoprotein expression or targeting eIF4a3-mediated regulatory pathways could be explored as potential therapeutic approaches in AML where GPR44 expression is low or not expressed [7]. Future studies should investigate the functional consequences of altered selenoprotein expression in *Gpr44*^−/−^ LICs, including their impact on cell survival, differentiation, and response to chemotherapy. Additionally, examining the role of prostaglandin signaling in selenium metabolism could provide further insights into the mechanisms underlying AML progression (Fig. 4).

In summary, our study provides evidence that GPR44 deletion alters the selenoproteome and selenoprotein synthesis in AML LICs, leading to increased leukemia aggressiveness. The observed changes in redox gatekeeper selenoproteins, coupled with upregulated eIF4a3 expression, suggest a complex regulatory network governing selenium metabolism in AML LICs. These findings open new avenues for understanding the role of selenium in leukemia progression and highlight potential targets for therapeutic intervention that involves specific targeting of LICs. Further studies are warranted to delineate the specific selenoproteins affected by this regulatory mechanism and to understand its implications beyond AML.

## Methods

### Protein Isolation and Immunoblotting Analysis in AML LICs

AML LICs were lysed using M-PER™ buffer (ThermoScientific) supplemented with 1% protease inhibitor cocktail (Sigma) and 25 mM sodium vanadate. Protein extraction was carried out according to the manufacturer’s protocol, and protein concentrations were determined using the Pierce™ BCA Protein Assay Kit (ThermoFisher). Extracted proteins were separated via standard sodium dodecyl sulfate–polyacrylamide gel electrophoresis (SDS-PAGE) and subsequently analyzed through Western blotting. The primary antibodies used for immunoblotting included rabbit anti-mouse Msrb1 (1:1000, Abclonal), rabbit anti-mouse Sps2 (1:1000, GeneTex), rabbit anti-mouse Gpx1 (1:1000, Abcam), rabbit anti-mouse Sep15 (Selenof) (1:1000, Abcam), rabbit anti-mouse Txnrd1 (1:2000, Proteintech), rabbit anti-mouse Selenom (1:1000, Epigenetex), rabbit anti-mouse Gpx2 (1:1000, Abclonal), rabbit anti-mouse Selenow (1:1000, Rockland), goat anti-mouse Selenoo (1:500, Santa Cruz biotechnology), rabbit anti-mouse Sbp2 (1:1000, Proteintech), and mouse anti-mouse β-actin (1:25,000, Fitzgerald). Protein expression levels were quantified using ImageJ (National Institutes of Health), with target protein expression normalized against β-actin.

### Quantification and Statistical Analysis

Statistical analyses were performed using GraphPad Prism version 6 (GraphPad Software), and data are expressed as mean ± SEM. Multiple *t*-test was used to compare two groups. A *p*-value of less than 0.05 was considered statistically significant, with significance levels denoted within each figure legend.

## Data Availability

No datasets were generated or analysed during the current study.
